# Safe zones of the maxillary alveolar bone in Down syndrome for orthodontic miniscrew placement assessed with cone-beam computed tomography

**DOI:** 10.1038/s41598-019-49345-0

**Published:** 2019-09-10

**Authors:** Jacobo Limeres Posse, María Teresa Abeleira Pazos, María Fernández Casado, Mercedes Outumuro Rial, Pedro Diz Dios, Márcio Diniz-Freitas

**Affiliations:** Medical-Surgical Dentistry Research Group (OMEQUI), Health Research Institute of Santiago de Compostela (IDIS), University of Santiago de Compostela (USC), Santiago de Compostela, Spain

**Keywords:** Oral anatomy, Orthodontics

## Abstract

The aim of this study was to quantify the available maxillary alveolar bone in a group of individuals with Down syndrome (DS) to determine the best areas for orthodontic miniscrew placement. The study group consisted of 40 patients with DS aged 12–30 years. We also selected an age and sex-matched control group. All measurements were performed on cross-sectional images obtained with cone-beam computed tomography. The selected areas of interest were the 4 interradicular spaces between the distal wall of the canine and the mesial wall of the second molar, in both maxillary quadrants. We measured the vestibular-palatine (VP) and mesiodistal (MD) dimensions to depths of 3, 6 and 9 mm from the alveolar ridge. We also measured the bone density in the same interradicular spaces of interest to 6 mm of depth from the alveolar crest. VP measurements were longer in the more posterior sectors and as the distance from the alveolar ridge increased. MD measurements also increased progressively as the distance from the alveolar ridge increased. In general, both the VP and MD measurements in the DS group were similar among the male and female participants. As age increased, the MD distance increased, while the VP distance decreased. The VP distance was ≥6 mm in at least 75% of the DS group in practically all assessed interdental spaces. The MD distance was ≥2 mm in at least 75% of the DS group only between the first and second molar, to 9 mm of depth from the alveolar ridge. The safe area for inserting orthodontic miniscrews in DS patients is restricted to the most posterior and deepest area of the maxillary alveolar bone.

## Introduction

Down syndrome (DS), also known as trisomy 21, was first described in 1866 by John Langdon Down and represents the most common live-born human aneuploidy. DS is associated with mental disability and has a characteristic phenotype in which orofacial abnormalities are common^1^.

It is estimated that more than 80% of individuals with DS have severe or highly severe dental malocclusions^2^. The demand for orthodontic treatment by this group has grown progressively^2^, although in many cases its approach can present a considerable challenge for the dental team^3^.

Technological advances, such as orthodontic temporary anchorage devices (TAD) that minimize the need for compliance for the success of dental movement techniques, can facilitate treatment in patients with special needs^4^. It has been suggested that palatine implants and miniplates have comparable or higher success rates than those achieved with miniscrews^5^. However, a recently published extensive review that analyzed 3250 miniscrews from 41 studies concluded that these provide a stable anchor for various dental movements and that its failure rate is acceptably low (13.5%)^6^. In the general population, miniscrews have been employed successfully for correcting skeletal abnormalities such as anterior open bite^7^, a frequent malocclusion in DS.

The advantages of miniscrews include their small size (which enables placement in numerous intraoral areas, including the interradicular spaces), low cost and the fact that both their insertion and withdrawal are simple procedures^8^. Additionally, their placement causes minimal trauma, and orthodontic traction force can be applied immediately or early compared with dental implants^9^.

In DS, miniscrews can be especially indicated because they reduce the degree of cooperation needed from the patient to achieve tooth movement and help solve the problem of crown size and periodontally affected teeth. When planning the insertion of miniscrews, it is essential to consider certain anatomical factors such as the proximity between the tooth roots, the available attached gingiva, the nerve structure topography, the maxillary sinus morphology and the cortical bone thickness^10^. The peculiarities of many of these anatomical structures have not yet been analyzed in individuals with DS. In the general population, the highest success rates for the miniscrews correspond to those placed in the midpalatal line, while the location with the poorest prognosis is the zygomatic buttress^11^. Alternative locations have been sought for inserting the miniscrews, such as the interradicular spaces between the upper molars^8^; however, the bone availability in this area in patients with DS is unknown. The aim of this study was to assess (in a group of individuals with DS) the topographical characteristics and bone density of the maxillary alveolar process in the locations where insertion of orthodontic miniscrews is most frequently recommended.

## Material and Methods

The study group (DS group) consisted of 40 white patients, selected according to the following inclusion criteria: age between 12 and 30 years; genetically confirmed diagnosis of DS; definitively erupted teeth; availability of three-dimensional cone-beam computed tomography (CBCT) images of acceptable quality of the maxilla; no history of surgical procedures in the maxillofacial area (with the exception of strictly dental procedures); and no previous orthodontic/orthopedic treatment or severe maxillary trauma.

The control group included 40 nonsyndromic individuals matched for age and sex. In addition to the selection criteria applied to the study group, we excluded those individuals with systemic disease that could affect orofacial development or dental/skeletal maturity directly or as a result of their treatment.

All images used in this study were from individuals with DS who had undergone upper and lower jaw CBCT in a previous study to evaluate atlantoaxial instability. The control group underwent CBCT mainly for the presurgical assessment of unerupted third molars, the root resorption assessment of periapical lesions, the assessment of temporomandibular (TMJ) disorders and cross-sectional imaging prior to dental implant placement. All images from the DS and control groups were retrieved from the archive of the Radiology Unit of the Faculty of Medicine and Dentistry at the University of Santiago de Compostela in Spain. All participants or, as applicable, their legal guardians signed an informed consent to authorize the use of images for teaching or research purposes. These radiological studies were performed in accordance with the radiation protection principles of “*As Low As Reasonably Achievable (ALARA)”* and following the guidelines of the SEDENTEXCT Guideline Development Panel, Radiation Protection No. 172: Cone Beam CT for Dental and Maxillofacial Radiology, Evidence Based Guidelines 2012 (www.sedentexct.eu). The study was approved by the ethics committee of the University of Santiago de Compostela.

All images employed in the study were obtained usnig an I-CAT® scanner (Imaging Sciences International, Hatfield, PA). The protocol for acquiring and manipulating the images has been previously described in detail^12^. All measurements were performed using open-source OsiriX image processing software (Pixmeo, Geneva, Switzerland; www.osirix-viewer.com).

**Figure 1:**
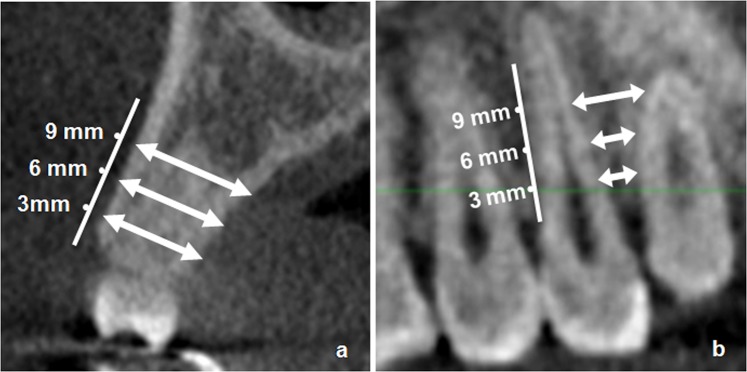
Measurement based on the cross-sectional cone-beam computed tomography images of the available maxillary alveolar bone at 3, 6 and 9 mm of depth from the alveolar ridge. (**a**) Vestibular-palatine dimension. (**b**) Mesiodistal dimension.

To perform the measurements in the vestibular-palatine (VP) direction based on the axial slices of the original CBCT images, we performed a panoramic reconstruction of the superior maxilla using the Dental3DPlugin® plugin. Using this panoramic reconstruction, we selected the areas of interest in both quadrants of the superior maxilla, which were the interradicular spaces from the distal wall of the canine to the mesial of the second molar. To associate data with a specific tooth, we employed the World Health Organization’s two-digit notation system (also known as ISO 3950 notation)^13^. From each area of interest, we obtained an image of a 1-mm thick cross-section with which we measured the distance between the external surfaces of the vestibular and palatal cortical bones of the superior maxilla, to depths of 3, 6 and 9 mm from the alveolar ridge (Fig. 1). We performed a total of 24 VP measurements per patient.

To perform the measurements in the mesiodistal (MD) direction, we used the same panoramic reconstruction of the superior maxilla described in the previous paragraph. Using this image, we selected the areas of interest and determined their interradicular distance to depths of 3, 6 and 9 mm from the alveolar ridge (Fig. 1), until a total of 24 MD measurements per patient had been completed.

In the event of missing teeth in the area of interest, we skipped the measurements in the corresponding interradicular spaces. In the event of pneumatization of the maxillary sinus with invasion of the interradicular space, the VP measurement was performed from the vestibular cortical bone of the superior maxilla to the vestibular wall of the sinus. When the dental roots that delimited an area of interest were shorter than 9 mm, we assigned the value of the maximum MD distance obtained in this area of interest to the interradicular distance corresponding to this depth from the alveolar ridge.

Once the VP and MD measurements had been performed, we delimited the ideal areas for the insertion of orthodontic miniscrews. To this end and based on the study by Martinelli *et al*.^14^, which recommended miniscrews with a diameter of 1.5–2.3 mm and a length of 6–8 mm, we established a minimum reference length of 6 mm in the VP direction and 2 mm in the MD direction. In each location, we defined 3 types of areas depending on the percentage of individuals who satisfied the criteria of minimum dimensions established as the reference: “safety area”, if at least 75% of the individuals met the criteria; “secondary area”, when the percentage was between 51% and 74%; and “risk area”, when the percentage did not exceed 50%.

To determine the bone density, we simulated the placement of a miniscrew in each of the interradicular locations of interest. To this end, we drew a rectangle (6 mm long by 1.5 mm wide) at a depth of 6 mm from the bone crest on the cross-sections of the superior maxilla obtained from the original CBCT images.

The statistical analysis was performed with the free R software (version 2.12.0, R Core Team, Vienna, Austria). The alveolar measurements MD and VP were recorded in millimeters and the density measurements were recorded using grayscale values. To evaluate intraobserver reliability, we randomly selected images from 10 participants (5 from the DS group and 5 from the control group) and a single trained observer performed all measurements during the study on 2 occasions with a 6-week interval. The reproducibility was evaluated using the intraclass correlation coefficient (ICC, 0.94; 95% confidence interval, 0.82–0.99). The significance for the differences between 2 sites within a single group (DS or control) was evaluated by performing multiple comparisons in a linear mixed model. We employed logistic regression to compare the values obtained from each site between the DS and control groups. Some of the variables did not follow a normal distribution. Therefore, to analyze the differences in the alveolar measurements evaluated according to sex in the DS and control groups, we employed the Wilcoxon test for independent samples. To analyze the differences in the measurements evaluated according to age, we calculated Spearman’s correlation coefficient. A p-value < 0.05 was considered statistically significant.

### Ethical approval

The study was approved by the Institutional Review Board of the University of Santiago de Compostela (USC), Spain.

### Informed consent

No specific informed consent was required as all participants or, as appropriate, their legal representatives had signed an informed consent to authorize the use of images for teaching or research purposes.

## Results

The DS group had a mean age of 17.8 ± 4.0 years, 15 were female, and 25 were male. The mean age of the control group was 17.5 ± 5.5 years and followed the same sex distribution.

The largest number of missing teeth in the DS group was detected in the anterior sector and mainly corresponded to the canines and right first premolars. For this reason, measurements for 10 patients could not be performed in this area.

### Measurements of the alveolar bone in the Down syndrome and control groups

The VP distance increased progressively (both in the DS and control groups) the further back the measurements were taken. In the DS group, the VP distance increased as the depth from the bone crest increased. In the control group, this tendency was not observed, and the opposite effect was observed in the intermolar spaces. The VP distance between the first and second molar was significantly shorter in the DS group than in the control group (Table 1).

**Table 1:** Vestibular-palatine (VP) dimensions at 3, 6 and 9 mm of depth from the bone crest in the Down Syndrome group and control group.

MEASUREMENT AREA		VP DIMENSION (mm)						
		Down syndrome			Control group			p value
Interdental space	Distance to bone crest	n	Mean ± SD	Range	n	Mean ± SD	Range	
13–14	3 mm	30	8.1 ± 1.6	5.0–14.2	33	8.2 ± 1.5	5.4–13.1	0.534
	6 mm		8.8 ± 1.8	5.3–15.5		8.7 ± 1.7	5.2–13.4	0.893
	9 mm		9.5 ± 2.2	4.9–17.1		9.0 ± 2.2	9.0–15.6	0.482
14–15	3 mm	32	8.7 ± 1.1	5.8–11.4	35	9.4 ± 1.2	7.0–13.1	0.010
	6 mm		9.0 ± 1.0	5.6–10.9		10.9 ± 1.4	6.5–13.4	0.455
	9 mm		9.4 ± 1.6	5.5–12.2		9.4 ± 1.9	4.0–14.0	0.745
15–16	3 mm	35	10.3 ± 1.3	7.1–12.5	39	11.1 ± 1.5	7.3–14.6	0.022
	6 mm		11.0 ± 1.8	6.0–13.8		11.2 ± 2.9	1.8–16.7	0.293
	9 mm		11.9 ± 2.1	6.0–14.8		10.4 ± 4.4	1.0–16.6	0.242
16–17	3 mm	39	11.5 ± 1.4	8.5–15.5	29	13.4 ± 1.1	11.2–16.1	0.000
	6 mm		11.9 ± 1.5	9.3–16.0		13.1 ± 3.2	0.9–17.3	0.000
	9 mm		10.6 ± 4.3	1.3–17.7		11.4 ± 5.7	0.7–18.2	0.050
23–24	3 mm	31	7.9 ± 1.5	5.1–12.1	33	8.3 ± 1.6	4.2–13.2	0.333
	6 mm		8.6 ± 1.7	5.6–13.7		8.6 ± 1.8	4.7–13.8	0.941
	9 mm		9.2 ± 2.0	5.9–15.3		9.0 ± 1.9	5.7–14.2	0.736
24–24	3 mm	32	8.8 ± 1.3	6.3–11.0	36	9.2 ± 1.2	6.4–12.5	0.512
	6 mm		9.1 ± 1.5	5.9–12.1		9.3 ± 1.3	6.2–13.1	0.912
	9 mm		11.0 ± 3.6	6.1–13.0		9.6 ± 1.4	7.2–13.5	0.661
25–26	3 mm	36	10.0 ± 1.5	6.7–13.1	38	11.2 ± 1.6	4.6–13.8	0.000
	6 mm		10.7 ± 1.7	7.3–14.3		11.6 ± 2.3	2.3–14.7	0.011
	9 mm		11.0 ± 3.6	1.3–16.2		10.5 ± 4.6	1.0–15.7	0.723
26–27	3 mm	38	11.8 ± 1.3	8.9–15.6	39	13.2 ± 1.3	9.9–15.9	0.000
	6 mm		11.9 ± 1.6	8.7–15.4		13.0 ± 3.5	1.5–16.8	0.000
	9 mm		11.0 ± 4.2	1.0–17.5		11.9 ± 5.5	1.1–19.7	0.014

VP, vestibular-palatine; n, number of participants; SD, standard deviation;13, maxillary right canine; 14, maxillary right first premolar; 15, maxillary right second premolar; 16, maxillary right first molar; 17, maxillary right second molar; 23, maxillary left canine; 24, maxillary left first premolar; 25, maxillary left second premolar; 26, maxillary left first molar; 27, maxillary left second molar.

The MD distance increased progressively (both in the DS and control groups) the farther the measurements were taken from the bone crest. The MD distance between the first and second molar measured at 6 and 9 mm of depth from the bone crest was significantly longer in the DS group than in the control group (Table 2).

**Table 2:** Mesiodistal (MD) dimensions at 3, 6 and 9 mm of depth from the bone crest in the Down Syndrome group and control group.

MEASUREMENT AREA		MD DIMENSION (mm)						
		Down syndrome			Control group			p value
Interdental space	Distance to bone crest	n	Mean ± SD	Range	n	Mean ± SD	Range	
13–14	3 mm	30	2.5 ± 1.4	1.2–7.0	33	2.9 ± 1.4	0.8–7.0	0.037
	6 mm		2.3 ± 0.9	0.6–4.6		3.0 ± 1.2	0.7–5.3	0.022
	9 mm		4.3 ± 2.3	0.4–7.0		3.9 ± 1.8	1.1–7.3	0.591
14–15	3 mm	32	2.7 ± 1.2	0.5–5.3	35	2.5 ± 0.8	1.0–4.3	0.674
	6 mm		2.9 ± 1.3	0.7–6.5		2.8 ± 1.0	0.9–5.0	0.960
	9 mm		4.5 ± 2.3	1.5–7.5		3.5 ± 2.0	0.9–7.5	0.050
15–16	3 mm	35	3.0 ± 1.2	0.8–6.6	39	2.8 ± 1.6	0.9–10.8	0.194
	6 mm		2.9 ± 1.6	0.5–8.0		3.1 ± 1.5	0.8–8.3	0.472
	9 mm		5.2 ± 3.7	0.7–10.7		4.9 ± 2.7	1.6–10.7	0.646
16–17	3 mm	39	2.1 ± 0.8	0.6–3.9	39	2.0 ± 0.9	0.7–4.1	0.465
	6 mm		2.6 ± 1.6	0.4–7.1		2.0 ± 1.6	0.3–7.1	0.043
	9 mm		4.7 ± 2.1	0.7–6.7		3.6 ± 2.5	0.2–6.7	0.021
23–24	3 mm	31	2.5 ± 1.0	0.6–4.9	33	2.5 ± 1.1	0.4–4.9	0.910
	6 mm		3.3 ± 1.9	1.0–7.8		2.4 ± 0.9	0.6–4.1	0.117
	9 mm		5.4 ± 2.6	0.8–7.8		3.4 ± 2.2	0.6–7.8	0.000
24–25	3 mm	32	2.6 ± 0.9	0.8–5.3	36	2.5 ± 1.0	0.6–5.3	0.726
	6 mm		2.6 ± 1.0	0.2–4.9		2.8 ± 1.2	0.8–6.0	0.882
	9 mm		3.8 ± 1.8	1.1–6.1		2.9 ± 1.4	6.1–6.1	0.050
25–26	3 mm	36	2.8 ± 1.2	0.9–7.1	38	3.1 ± 1.5	1.1–7.1	0.472
	6 mm		2.8 ± 1.5	0.4–8.2		3.2 ± 1.8	0.5–8.2	0.343
	9 mm		4.8 ± 3.4	0.6–10.3		4.8 ± 2.8	0.5–10.3	0.615
26–27	3 mm	38	2.0 ± 0.9	0.7–4.3	39	1.9 ± 0.9	0.5–4.7	0.764
	6 mm		2.4 ± 1.3	0.6–5.7		1.9 ± 1.8	0.3–7.1	0.027
	9 mm		4.0 ± 1.6	0.3–5.7		3.0 ± 1.9	0.4–5.7	0.023

MD, mesiodistal; n, number of participants; SD, standard deviation; 13, maxillary right canine; 14, maxillary right first premolar; 15, maxillary right second premolar; 16, maxillary right first molar; 17, maxillary right second molar; 23, maxillary left canine; 24, maxillary left first premolar; 25, maxillary left second premolar; 26, maxillary left first molar; 27, maxillary left second molar.

### Measurements of the alveolar bone in relation to sex

In general, the VP distances were similar in the male and female patients in the DS group. In the control group, the VP distances between the canine and first premolar and between the first and second premolar were significantly longer in the male patients than in the female patients (Table 3).

**Table 3:** Vestibular-palatine (VP) dimensions at 3, 6 and 9 mm of depth from the bone crest in the Down Syndrome group and control group, according to sex.

MEASUREMENT AREA		VP DIMENSION (mm)									
		Down syndrome					Control group				
		Male		Female		p value	Male		Female		p value
Interdental space	Distance to bone crest	n	Mean ± SD	n	Mean ± SD		n	Mean ± SD	n	Mean ± SD	
13–14	3 mm	19	8.1 ± 1.7	11	7.9 ± 1.4	0.999	22	8.5 ± 1.5	11	7.6 ± 1.4	0.143
	6 mm		9.0 ± 1.9		8.4 ± 1.5	0.401		9.2 ± 1.6		7.6 ± 1.5	0.015
	9 mm		9.9 ± 2.3		8.7 ± 2.0	0.103		9.6 ± 2.1		7.7 ± 1.8	0.022
14–15	3 mm	18	8.7 ± 0.8	14	8.7 ± 1.5	0.732	22	9.8 ± 1.3	13	8.9 ± 0.9	0.031
	6 mm		9.1 ± 1.1		8.8 ± 1.6	0.704		9.7 ± 1.5		8.8 ± 1.2	0.094
	9 mm		9.7 ± 1.4		9.0 ± 1.9	0.332		10.0 ± 1.8		8.4 ± 1.9	0.042
15–16	3 mm	20	10.3 ± 1.0	15	10.3 ± 1.7	0.526	25	11.0 ± 1.5	14	11.3 ± 1.7	0.319
	6 mm		11.1 ± 1.4		10.9 ± 2.2	0.802		11.7 ± 1.8		10.3 ± 4.1	0.608
	9 mm		12.0 ± 2.0		11.7 ± 2.4	0.92		11.1 ± 3.6		9.1 ± 5.4	0.334
16–17	3 mm	24	11.5 ± 1.4	15	11.6 ± 1.4	0.665	26	13.5 ± 1.2	13	13.1 ± 0.9	0.325
	6 mm		11.8 ± 1.5		12.1 ± 1.5	0.443		13.5 ± 2.3		12.2 ± 4.4	0.612
	9 mm		11.2 ± 3.7		9.7 ± 5.2	0.772		11.5 ± 5.6		11.3 ± 6.1	0.976
23–24	3 mm	20	8.3 ± 1.5	12	7.2 ± 1.1	0.044	21	8.9 ± 1.5	12	7.2 ± 1.2	0.002
	6 mm		9.1 ± 1.6		7.8 ± 1.5	0.056		9.5 ± 1.6		7.1 ± 1.1	0.000
	9 mm		9.9 ± 2.0		8.1 ± 1.6	0.014		9.7 ± 1.9		7.7 ± 1.4	0.004
24–25	3 mm	19	8.8 ± 1.2	14	8.8 ± 1.5	0.743	22	9.7 ± 1.1	14	8.4 ± 0.9	0.001
	6 mm		9.1 ± 1.3		9.0 ± 1.7	0.956		9.9 ± 1.2		8.3 ± 0.9	0.001
	9 mm		9.9 ± 1.4		9.3 ± 1.9	0.635		10.1 ± 1.5		8.8 ± 1.0	0.016
25–26	3 mm	21	10.0 ± 1.1	15	10.1 ± 1.9	0.7	25	11.5 ± 1.3	13	10.6 ± 2.1	0.248
	6 mm		10.7 ± 1.5		10.7 ± 2.1	0.7		12.0 ± 1.7		10.8 ± 3.1	0.397
	9 mm		10.6 ± 3.7		11.4 ± 3.5	0.451		11.5 ± 4.0		8.6 ± 5.3	0.090
26–27	3 mm	23	11.6 ± 1.2	15	11.9 ± 1.5	0.324	26	13.5 ± 1.2	13	12.6 ± 1.3	0.107
	6 mm		11.7 ± 1.6		12.1 ± 1.5	0.437		14.0 ± 2.0		11.2 ± 5.0	0.107
	9 mm		11.4 ± 3.7		10.2 ± 4.9	0.591		12.4 ± 5.4		11.0 ± 5.7	0.531

VP, vestibular-palatine; n, number of participants; SD, standard deviation; 13, maxillary right canine; 14, maxillary right first premolar; 15, maxillary right second premolar; 16, maxillary right first molar; 17, maxillary right second molar; 23, maxillary left canine; 24, maxillary left first premolar; 25, maxillary left second premolar; 26, maxillary left first molar; 27, maxillary left second molar.

There were no statistically significant differences in the MD distances between the male and female patients, either in the DS or control group, except in specific cases (e.g., the MD distance at 3 mm of depth from the bone crest between the second premolar and first molar was longer in the female patients than in the male patients of the DS group) (Table 4).

**Table 4:** Mesiodistal (MD) dimensions at 3, 6 and 9 mm of depth from the bone crest in the Down Syndrome group and control group, according to sex.

MEASUREMENT AREA		MD DIMENSION (mm)									
		Down syndrome					Control group				
		Male		Female		p value	Male		Female		p value
Interdental space	Distance to bone crest	n	Mean ± SD	n	Mean ± SD		n	Mean ± SD	n	Mean ± SD	
13–14	3 mm	19	2.3 ± 0.8	11	2.8 ± 2.0	0.964	22	2.9 ± 1.6	11	2.9 ± 0.9	0.721
	6 mm		2.4 ± 1.0		2.0 ± 0.6	0.238		2.9 ± 1.2		3.0 ± 1.2	0.702
	9 mm		4.8 ± 2.4		3.4 ± 2.0	0.133		3.8 ± 1.9		4.1 ± 1.7	0.833
14–15	3 mm	18	2.7 ± 0.8	14	2.7 ± 1.6	0.382	22	2.4 ± 0.7	13	2.6 ± 0.8	0.533
	6 mm		3.1 ± 1.2		2.6 ± 1.5	0.193		2.7 ± 1.0		3.0 ± 1.1	0.597
	9 mm		4.6 ± 2.2		4.4 ± 2.5	0.642		3.2 ± 1.9		4.0 ± 2.2	0.313
15–16	3 mm	20	2.6 ± 0.8	15	3.5 ± 1.4	0.027	25	2.9 ± 1.8	14	2.6 ± 1.3	0.826
	6 mm		2.6 ± 1.3		3.3 ± 1.9	0.182		3.2 ± 1.4		3.0 ± 1.8	0.292
	9 mm		4.6 ± 3.4		6.0 ± 4.1	0.458		4.6 ± 2.4		5.3 ± 3.2	0.837
16–17	3 mm	24	2.1 ± 0.7	15	2.1 ± 1.0	0.869	26	2.1 ± 1.0	13	1.7 ± 0.8	0.340
	6 mm		2.3 ± 1.1		3.1 ± 2.2	0.565		2.0 ± 1.5		1.9 ± 1.9	0.712
	9 mm		4.5 ± 2.0		5.0 ± 2.2	0.512		3.8 ± 2.5		3.2 ± 2.4	0.412
23–24	3 mm	20	2.4 ± 1.1	12	2.6 ± 0.9	0.425	21	2.1 ± 1.1	12	3.1 ± 0.9	0.022
	6 mm		3.3 ± 1.7		3.2 ± 2.2	0.349		2.3 ± 1.0		2.7 ± 0.7	0.166
	9 mm		5.7 ± 2.6		5.0 ± 2.6	0.441		3.4 ± 2.4		3.3 ± 1.9	0.793
24–25	3 mm	19	2.3 ± 0.7	14	3.0 ± 1.1	0.053	22	2.5 ± 1.0	14	2.6 ± 0.9	0.398
	6 mm		2.6 ± 1.0		2.6 ± 1.0	0.785		2.6 ± 1.2		3.0 ± 1.1	0.153
	9 mm		3.8 ± 1.7		3.9 ± 1.9	0.985		2.7 ± 1.5		3.2 ± 1.3	0.269
25–26	3 mm	21	2.3 ± 0.9	15	3.4 ± 1.4	0.01	25	3.2 ± 1.7	13	2.8 ± 1.2	0.548
	6 mm		2.6 ± 1.7		3.1 ± 1.1	0.133		3.0 ± 1.6		3.5 ± 2.2	0.902
	9 mm		4.0 ± 3.4		6.0 ± 3.3	0.026		4.1 ± 2.5		6.1 ± 3.0	0.030
26–27	3 mm	23	1.9 ± 0.9	15	2.1 ± 1.0	0.742	26	2.0 ± 1.0	13	1.8 ± 0.7	0.905
	6 mm		2.3 ± 1.2		2.5 ± 1.4	0.79		2.0 ± 1.5		1.9 ± 2.3	0.185
	9 mm		4.1 ± 1.5		4.0 ± 1.9	0.999		3.1 ± 2.0		2.8 ± 1.9	0.558

MD, mesiodistal; n, number of participants; SD, standard deviation; 13, maxillary right canine; 14, maxillary right first premolar; 15, maxillary right second premolar; 16, maxillary right first molar; 17, maxillary right second molar; 23, maxillary left canine; 24, maxillary left first premolar; 25, maxillary left second premolar; 26, maxillary left first molar; 27, maxillary left second molar.

### Measurements of the alveolar bone in relation to age

In terms of the VP distance, all Spearman correlations were negative, which implies that the VP distance decreased as age increased. In the DS group, this finding was verified in virtually all evaluated interdental spaces, while in the control group, this finding was limited to posterior sectors (between the second premolar and first molar and between the first and second molar) (Table 5).

**Table 5:** Vestibular-Palatine (VP) and Mesiodistal (MD) dimensions at 3, 6 and 9 mm of depth from the bone crest in the Down Syndrome group and control group, according to age.

MEASUREMENT AREA		VP DIMENSION (mm)				M-D DIMENSION (mm)			
		Down syndrome		Control group		Down syndrome		Control group	
Interdental space	Distance to bone crest	SCC	p value	SCC	p value	SCC	p value	SCC	p value
13–14	3 mm	−0.44	0.006	−0.21	0.799	−0.28	0.000	−0.23	0.000
	6 mm	−0.42	0.064	−0.16	0.921	0.46	0.000	−0.23	0.000
	9 mm	−0.24	0.409	−0.09	0.830	0.38	0.001	−0.06	0.000
14–15	3 mm	−0.46	0.000	−0.38	0.002	−0.15	0.002	0.2	0.173
	6 mm	−0.52	0.000	−0.5	0.000	0.32	0.07	0.18	0.094
	9 mm	−0.35	0.000	−0.5	0.000	0.47	0.001	0.13	0.139
15–16	3 mm	−0.47	0.000	−0.54	0.000	−0.1	0.096	0.27	0.012
	6 mm	−0.52	0.000	−0.72	0.000	0.35	0.012	0.48	0.000
	9 mm	−0.58	0.000	−0.43	0.000	0.42	0.002	0.42	0.000
16–17	3 mm	−0.49	0.000	−0.41	0.000	0.39	0.024	0.42	0.000
	6 mm	−0.5	0.000	−0.45	0.000	0.22	0.42	0.39	0.000
	9 mm	−0.46	0.000	−0.49	0.000	0.31	0.009	0.44	0.000
23–24	3 mm	−0.39	0.007	−0.03	0.163	−0.29	0.000	−0.27	0.000
	6 mm	−0.37	0.026	−0.01	0.246	0.14	0.191	0.03	0.040
	9 mm	−0.39	0.01	−0.1	0.544	0.48	0.000	0.08	0.235
24–25	3 mm	−0.68	0.000	−0.15	0.37	−0.15	0.002	−0.3	0.000
	6 mm	−0.7	0.000	−0.17	0.213	0.45	0.001	−0.18	0.000
	9 mm	−0.53	0.000	−0.21	0.053	0.44	0.000	0.04	0.114
25–26	3 mm	−0.61	0.000	−0.45	0.000	0.04	0.314	0.08	0.421
	6 mm	−0.62	0.000	−0.52	0.000	0.37	0.006	0.36	0.016
	9 mm	−0.57	0.000	−0.56	0.000	0.43	0.000	0.26	0.009
26–27	3 mm	−0.6	0.000	−0.33	0.000	0.42	0.039	0.35	0.000
	6 mm	−0.58	0.000	−0.32	0.000	0.41	0.031	0.54	0.000
	9 mm	−0.49	0.000	−0.6	0.000	0.51	0.005	0.37	0.000

VP, vestibular-palatine; SCC, Spearman’s correlation coefficient; SD, standard deviation; 13, maxillary right canine; 14, maxillary right first premolar; 15, maxillary right second premolar; 16, maxillary right first molar; 17, maxillary right second molar; 23, maxillary left canine; 24, maxillary left first premolar; 25, maxillary left second premolar; 26, maxillary left first molar; 27, maxillary left second molar.

When assessing the MD distance, in contrast, most of the Spearman correlations were positive, which implies that the MD distance also increased with age. This finding was confirmed in the DS and control groups, except in specific cases (e.g., between the right first and second premolar) (Table 5).

### Ideal areas for inserting orthodontic miniscrews

**Figure 2:**
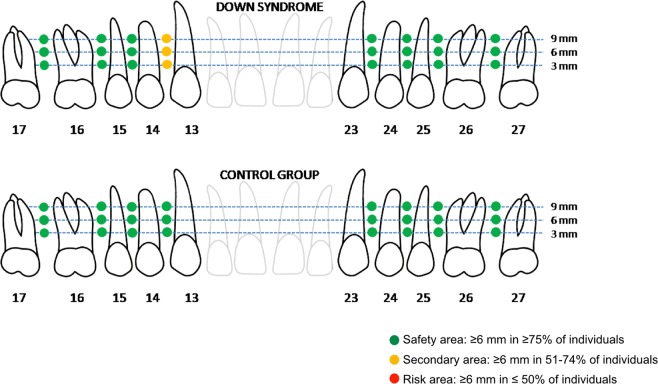
Determining the ideal areas for orthodontic miniscrew placement based on the vestibular-palatine dimension of the available maxillary alveolar bone.

Based on the VP measurements, all evaluated interdental spaces can be considered “safe areas” (in the DS and control groups), except between the canine and right first premolars in the DS group (which is a “secondary area”) (Fig. 2).

**Figure 3:**
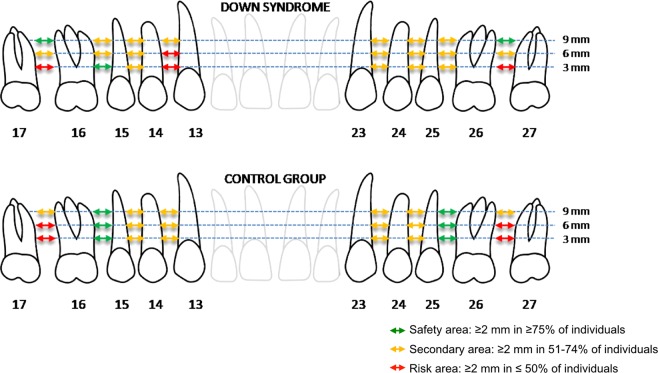
Determining the ideal areas for orthodontic miniscrew placement based on the mesiodistal dimension of the available maxillary alveolar bone.

Based on the MD measurements, the only bilateral “safe area” in the DS group is between the first and second molar, at 9 mm of depth from the bone crest. In the control group, the only “safe area” was between the second premolar and first molar, at 3, 6 or 9 mm of depth from the bone crest (Fig. 3).

### Bone density measurement in the Down syndrome and control groups

In general, there were no significant differences between the participants with DS and the control group, except in the interradicular space between the first and second upper right molar, where the bone density was significantly lower in the participants with DS (Table 6).

**Table 6:** Bone density at a depth of 6 mm from the bone crest in the Down Syndrome group and in the control group.

MEASUREMENT AREA	BONE DENSITY (GV)						
	Down syndrome			n	Control group		p value
Interdental space	n	Mean ± SD	Range		Mean ± SD	Range	
13–14	30	817.51 ± 285.90	233.76–1481.24	33	654.03 ± 306.97	70.93–1253.80	0.062
14–15	32	545.12 ± 277.74	49.39–1061.48	35	632.90 ± 231.04	241.75–1016.88	0.243
15–16	35	544.01 ± 325.91	16.93–1307.72	37	564.39 ± 261.75	19.58–1017.17	0.817
16–17	38	470.44 ± 210.30	4.86–1217.25	36	683.91 ± 374.03	13.86–1329.44	0.040
23–24	32	764.56 ± 263.62	384.16–1103.35	33	747.46 ± 301.34	37.09–1328.30	0.821
24–24	33	660.45 ± 274.23	255.77–1244.23	36	674.08 ± 318.41	47.48–1262.00	0.879
25–26	36	584.43 ± 274.23	225.01–1278.38	37	673.99 ± 346.56	42.11–1414.94	0.235
26–27	37	492.46 ± 278.79	65.66–1033.24	35	649.19 ± 288.21	128.10–1289.26	0.065

GV, grey values; n, number of participants; SD, standard deviation; 13, maxillary right canine; 14, maxillary right first premolar; 15, maxillary right second premolar; 16, maxillary right first molar; 17, maxillary right second molar; 23, maxillary left canine; 24, maxillary left first premolar; 25, maxillary left second premolar; 26, maxillary left first molar; 27, maxillary left second molar.

When studying the influence of sex on the bone density values in the study group, we found no significant differences between the male and female patients, except in the interradicular spaces between the first and second upper right premolar and between the first and second upper right molar, where the bone density was significantly greater in the female patients than in the male patients (Table 7).

**Table 7:** Bone density at a depth of 6 mm from the bone crest in the Down Syndrome group and in the control group, by sex.

MEASUREMENT AREA	BONE DENSITY (GV)									
	Down syndrome					Control group				
	Male		Female		p value	Male		Female		p value
Interdental space	n	Mean ± SD	n	Mean ± SD		n	Mean ± SD	n	Mean ± SD	
13–14	19	802.70 ± 333.25	11	838.24 ± 218.30	0.772	21	617.43 ± 339.80	12	742.91 ± 200.77	0.371
14–15	18	421.54 ± 230.19	14	718.15 ± 252.14	0.013	21	620.09 ± 234.07	14	663.99 ± 238.62	0.683
15–16	20	468.60 ± 286.50	15	649.60 ± 320.08	0.168	24	542.92 ± 292.52	13	616.52 ± 173.12	0.542
16–17	23	335.63 ± 246.87	15	659.18 ± 339.93	0.012	24	580.46 ± 373.51	12	935.16 ± 245.53	0.032
23–24	20	726.19 ± 205.77	12	818.29 ± 215.31	0.308	20	717.13 ± 315.78	13	821.11 ± 270.65	0.456
24–25	19	580.86 ± 183.48	14	771.87 ± 324.53	0.088	21	710.68 ± 293.91	15	585.19 ± 381.16	0.397
25–26	21	541.01 ± 248.21	15	645.23 ± 310.08	0.374	24	658.12 ± 369.17	13	712.54 ± 307.47	0.739
26–27	22	402.04 ± 216.58	15	619.06 ± 316.77	0.062	24	582.90 ± 267.65	11	810.20 ± 291.00	0.082

GV, grey values; n, number of participants; SD, standard deviation; 13, maxillary right canine; 14, maxillary right first premolar; 15, maxillary right second premolar; 16, maxillary right first molar; 17, maxillary right second molar; 23, maxillary left canine; 24, maxillary left first premolar; 25, maxillary left second premolar; 26, maxillary left first molar; 27, maxillary left second molar.

In general, age did not affect the bone density values, except for the interradicular spaces between the second premolar and the first upper right molar (p = 0.01) and between the canine and the first upper left premolar (p = 0.03).

## Discussion

A particularly narrow morphology of the palate but with anteroposterior dimensions and vault height similar to those of the general population is considered a phenotypic characteristic of DS^15^. In specific locations such as the midpalatal suture area, the palatal bone is also thinner in patients with DS than in nonsyndromic controls paired by age and sex. We can therefore expect that there are other morphometric characteristics in the maxillary area that are inherent to the syndrome, although we have thus far found no published studies to confirm it.

The present study is not exempt from a number of methodological limitations that should be considered when extrapolating our results. The participants’ age range is much wider than that of participants in previous studies that used a similar methodology^16^. All participants were white; however, it has been indicated that facial morphometry is determined by ethnicity and race^17^. In any case, the diameter and length of the miniscrew implants does not ensure the success of the procedure^18^.

In the DS group, the longest VP distances were detected in the intermolar area, which allows for the insertion of longer miniscrews in this area. It has been shown that, in the general population, the thickest part of the bone in the buccopalatal dimension in the maxillary arch is also between the first and second molars^8^. The available bone in the intermolar area was thinner in the DS group than in the control group, a result that could be explained by the fact that the hard palate is narrower in the DS group than in the control group^19^.

The diameter of the selected miniscrew will be determined by the MD length of available bone. It has been suggested that skeletal anchoring of the miniscrews is stable when they have a diameter between 1.5 and 2.3 mm^7^. The MD distance is highly relevant, given that one of the greatest risks for miniscrew failure is its proximity to the teeth roots^20^. In the DS group, the intermolar area presented a longer MD distance, and the amount of available MD bone was greater than in the control group, probably because the teeth of the DS group were smaller than those of the control group. Microdontia is not uncommon among patients with DS and can result in spacing^21^. In the general population, the intermolar area is also considered one of the most reachable locations for miniscrew placement because it has a larger space between the tooth roots^20^. A number of authors have indicated that the longest MD distance in the maxilla is on the palatal side between the second premolar and first molar^8^.

In the DS group, we found no significant differences in length for the maxillary alveolar bone between the male and female patients. This finding is consistent with our previous results in DS in which sex did not affect the morphometry of the hard palate^15^ or the palatal bone thickness^12^. In contrast, a number of measurements in the canine and premolar areas were larger in the male patients than in the female patients in the control group, confirming the sexual dimorphism detected in the general population in most craniofacial measurements from childhood^22^, in the dimensions of the hard palate^15^ and in the cortical bone thickness in the maxillary posterior region^23^.

We observed a significant reduction in the VP distance with age in both the DS and control groups, which could be the result of a slight decrease in the dental arch widths reported after the complete eruption of permanent dentition^24^. In contrast, the available bone in the MD direction increased with age, probably due to the significant effect that age has on anteroposterior palatal length^15^.

In the DS group and based on the MD measurements, the “safe area” corresponded to the deepest locations of the intermolar space. In the general population, it has been shown that cortical bone density and thickness significantly increase from the coronal to the apical regions of the alveolar bone^25^, and the most favorable areas for miniscrew insertion in the maxilla have been proposed from the second premolar to the second molar^26^.

A number of studies have shown reduced bone mass in children and adolescents with DS^27^. An important recently published series found that the maximum peak bone mass was reached earlier than in the general population but that those levels were lower than in the general population^28^. Additionally, individuals with DS have a number of risk factors that can promote the onset of osteoporosis, such as premature aging, developmental disorders, physical inactivity, limited sun exposure, comorbidities and drug consumption that can alter bone metabolism. In this study, we detected no significant differences in the bone density of the upper maxilla between the participants with DS and the control group. We have found no similar studies in the literature with which to compare these results. In any case, it seems that the main risk factor for the failure of orthodontic miniscrews is the proximity of the miniscrew to the tooth roots^11^, while bone density does not appear to have a determinant effect^29^.

When determining the best location for placing miniscrews, panoramic radiographs can underestimate the available interdental bone space^30^. The accuracy of periapical radiographs is greater than that of panoramic radiographs when assessing the length of the tooth roots^31^ and when determining the position of mini-implants^32^. CBCT, is superior to periapical radiographs when assessing the proximity of the miniscrews to the tooth roots, and its application has increased the success of this orthodontic anchoring technique^29^. In general, 2D radiographs enable an approximate assessment of the miniscrew’s position relative to the surrounding structures, especially when maps of “safe zones” are available, such as those of the present study. CBCT should be reserved for borderline cases in which the interradicular distance or the quantity of available bone tissue in the VP direction is uncertain.

In conclusion, “safe areas” for insertion of orthodontic miniscrews in individuals with DS are limited, especially by the MD distance of the available maxillary alveolar bone.

## Data Availability

The datasets generated during and/or analyzed during the current study are available from the corresponding author on reasonable request.
